# Global Self-Esteem, Body Composition, and Physical Activity in Polish University Students

**DOI:** 10.3390/nu15183907

**Published:** 2023-09-08

**Authors:** Karolina Krupa-Kotara, Jarosław Markowski, Agnieszka Gdańska, Mateusz Grajek, Eliza Działach, Grzegorz Szlachta, Mateusz Rozmiarek

**Affiliations:** 1 Department of Epidemiology, Department of Epidemiology and Biostatistics, Faculty of Public Health in Bytom, Silesian Medical University in Katowice, 40-055 Katowice, Poland; 2 Department of Laryngology, Faculty of Medical Sciences, Silesian Medical University in Katowice, 40-055 Katowice, Poland; jmarkowski@sum.edu.pl; 3 Department of Public Health, Department of Public Health Policy, Faculty of Public Health in Bytom, Silesian Medical University in Katowice, 40-055 Katowice, Poland; a.gdanska@awf.katowice.pl (A.G.); mgrajek@sum.edu.pl (M.G.); eliza.dzialach@gmail.com (E.D.); 4 Motion Analysis Laboratory, Institute of Physiotherapy and Health Sciences, The Jerzy Kukuczka Academy of Physical Education, 40-065 Katowice, Poland; g.szlachta@awf.katowice.pl; 5 Department of Sports Tourism, Faculty of Physical Culture Sciences, Poznan University of Physical Education, 61-871 Poznan, Poland; rozmiarek@awf.poznan.pl

**Keywords:** global self-esteem, physical activity, body composition, students, self-esteem

## Abstract

Global self-esteem is a psychological concept that refers to the overall assessment of oneself as a person of value. Body composition is measured by indices such as BMI, BF, and LBM, which have implications for health and self-esteem. Physical activity is associated with numerous health and self-esteem benefits. The purpose of this study focuses on assessing the relationship between global self-esteem, body composition, and physical activity among Polish students. The study was conducted on a group of 305 students between the ages of 19 and 26. The participants were divided into groups according to their level of physical activity. The SES scale by M. Rosenberg assessed the subjects’ global self-esteem. The results showed a negative correlation between body weight and BMI and global self-esteem, that is, the higher the body weight, the lower the self-esteem. On the other hand, higher levels of physical activity were associated with higher global self-esteem. The study suggests that maintaining healthy physical activity and body composition can help improve global self-esteem. The study highlights the importance of physical activity for mental health and self-esteem. It is worth encouraging and promoting physical activity among students to support an individual’s physical and emotional health. Ultimately, this study may have implications for health policy, education, and intervention for students to emphasize the importance of physical activity for self-esteem and overall health.

## 1. Introduction

Self-esteem in psychology is a relatively stable attitude toward one’s own “self”, which is based on comparing one’s person (behavior, physical or mental characteristics) with a model, which is considered the so-called “ideal self” [1]. Self-esteem consists of several elements among which are self-esteem, satisfaction with one’s appearance, or health. These elements are closely interrelated and depend on social, cultural, or environmental factors [2].

Related to self-esteem is the concept of so-called global self-esteem, which has been defined as a general assessment of oneself as a person of value [3]. Global self-esteem is associated with several elements of self-perception, notable among which are body image and self-esteem. These can affect changes in the perception of global self-esteem [4]. High self-esteem is associated with high global self-evaluation; conversely, low self-esteem refers to unfavorable self-evaluation [5] (Baumeister et al., 2003). Similarly, satisfaction with one’s appearance refers to general aspects of body satisfaction, while dissatisfaction refers to negative subjective evaluations of one’s body [6].

People’s level of self-esteem varies at different stages of life and is related to life experiences and feelings of acceptance in one’s environment. Self-esteem can affect many elements of life, although scientific research on this topic provides unclear results [3]. Self-esteem changes stages of life; in particular, these changes are observed in adolescence and late adulthood. This is particularly the case during those periods of life in which there are changes in the figure or physique resulting from puberty or the aging process. The effect of dissatisfaction with one’s body can lower self-esteem and can be a risk factor for self-esteem. Changes in body shape and figure are associated with changes in body weight and may depend on factors such as physical activity and diet, among others [7,8].

Many indices are used to monitor body weight, among which are BMI, WHR, or body composition assessment. The body mass index (BMI = weight/height^2^) is often used to assess body weight, even though by definition it does not distinguish between its components such as levels of body fat or muscle mass [8]. The second component used in assessing nutritional status is body composition assessment, which includes the measurement of body fat and lean mass—the components of body mass. It is mainly used to monitor training performance in a sports environment and for general verification of population health [9].

The use of BMI as a predictor of obesity and the resulting health risk should be used with caution, especially for physically active people, who tend to have higher lean body mass (LBM) than the general population [10]. However, body fat levels are strongly correlated with increased mortality, with the risk of diseases of civilization, among which are cardiovascular and metabolic diseases. In the case of these diseases, the elements used for prevention include physical activity and proper eating habits, as well as maintaining a normal body weight [11].

Physical activity is associated with various physical and mental health benefits across all population groups. Additionally, it is associated with developing self-esteem and self-worth, but the relationship remains unclear. The benefits of physical activity have been repeatedly documented in many mental and physical health studies [12]. Despite these benefits, many people in society do not adhere to recommended levels of physical activity [13]. On the contrary, some people play sports professionally or exercise more intensively. In this group, we can distinguish between those who exercise for health reasons to improve their well-being and those who exercise mainly to care for their physical appearance. We are not sure how related measures of physical health, such as sedentary lifestyle, body composition, and physical fitness, affect the relationship between physical activity and self-esteem [14].

Given this rationale, this study investigated whether students’ global self-esteem is related to their level of physical activity and whether these relationships may be related to different motivations for training.

## 2. Materials and Methods

### 2.1. Characteristics of the Study Group

The study group included 305 undergraduate students aged 19–26 years (22.28 ± 1.85 years), including 192 (63%) women and 113 (37%) men. The students were full-time students and studied at universities located in Katowice (Silesian Voivodeship, Poland). The students were at different stages of education. A total of 153 people (50.16%) were at the stage of the first degree, 119 people (39.01%) were at the stage of the second degree, and 33 people (10.8%) comprised the remaining participants of the study who were a group of people attending doctoral studies, or third-degree studies.

### 2.2. Eligibility Criteria for Participants

Exclusion criteria for the study were age < 18 years and >26 years, lack of full-time student status, and diseases or factors that may limit daily physical activity, such as musculoskeletal dysfunction, cardiovascular disease, diabetes, and hypertension. Participation in the study was voluntary. Participants were informed about the purpose and conduct of the study, as well as the possibility of opting out at any stage.

The research was carried out in strict adherence to ethical guidelines, ensuring the highest standards of integrity and respect for the rights of participants. Specifically, the study conforms to the stipulations laid out in the Helsinki Declaration, a landmark document that provides principles and guidelines for ethical considerations concerning human participants in medical research. Furthermore, to ensure that the research meets local standards, it also secured approval from the Bioethical Commission of the Silesian Medical University in Katowice. This commission serves as a regulatory body that oversees and ensures that all research undertaken complies with the accepted ethical standards and protects the rights and well-being of participants. The specific approval ID for this research is PCN/CBN/0052/KB/127/22, which can be referenced for further verification and transparency. This underscores the commitment of the researchers to undertake the study with the utmost ethical consideration and with respect for both international and local ethical guidelines.

### 2.3. Test Procedure

The study group included people declaring a low level of physical activity (PAL > 5000 steps per day); a medium level of physical activity (PAL 5000–10,000 steps per day), and people with a high level of physical activity (PAL > 10,000), including professional athletes representing AZS (Academic Sports Association) or I and II league sports clubs from the areas of the Silesian Province.

In all study groups, the level of physical activity, the type and volume of training per week, and the level of global self-esteem were assessed. Anthropometric measurements (body height, body weight) were taken in the students studied and the following body mass indexes were determined:

- The level of body fat BF (kg) and lean mass LBM (kg) and their percentage (%BF, %LBM) measured by electrical bioimpedance (Tanita TBF-300M),
- BMI (Body Mass Index) indices with the adoption of the following norms: BMI 17–18.49 kg/m^2^—underweight; 24.9–29.9 kg/m^2^—overweight; 30 < obesity (WHO 2018) and WHR ≥ 0.80 (WHO 2011).

To assess physical activity, an objective tool was used—wristbands equipped with XIAOMI Mi Band 5 and XIAOMI Mi Band 6 pedometers. For physical activity assessed with pedometers, norms were adopted according to Oliveira et al. (2019): <5000 steps/day—sedentary lifestyle; 5000–7499—low physical activity; 7500–9999 steps/day—moderate physical activity; >10,000 steps/day—desirable physical activity. Pedometers estimated the weekly number of steps. The wristbands were removed only for bathing and sleeping.

### 2.4. Tools Used

In the students surveyed, the level of global self-esteem was assessed using a questionnaire containing the SES scale by M. Rosenberg in a Polish adaptation by Łaguna, the utility of which has been demonstrated in a number of studies [15,16,17,18]. Participants were asked to complete the questionnaire after being weighed and assessed for physical activity. The Rosenberg scale is a tool for assessing global self-esteem, understood as an attitude toward oneself. All statements on the scale are scored from 1 to 4—from strongly agree to strongly disagree. The sum of points obtained when completing the questionnaire is an indicator of its level. The range of points possible is from 10 to 40, which is distinguished as follows: 10–27 points—low self-esteem; 28–32 points—medium self-esteem; 33–40 points—high self-esteem. In some versions of the scale, scoring occurs in the 0–3 range, which may account for other point ranges [19,20].

### 2.5. Statistical Analysis

The study used Shapiro–Wilk and Spearman analytical tests to verify the hypotheses. The probability level was set as 0.05.

## 3. Results

In the study group, the average body weight was 71.69 ± 13.72 kg, while the height of the subjects was 1.69 m. Based on the measurement of body weight and height, the value of the BMI index was calculated. The value of this index means 18.5–24.99—normal weight 25.0–29.9—overweight 30.0–34.99—grade I obesity. 35.0–39.99—grade II obesity; the study group was characterized by the presence of overweight. The mean BMI value was 25.14 ± 4.22 (Table 1).

Average self-esteem was observed in the study group. The score obtained in the study group was 29.2 ± 13.72. Interpretation of the index was as follows: 10–27 points indicate low self-esteem, 28–30 points are average self-esteem, and 32–40 points are high self-esteem (Table 2).

In the study group, physical activity calculated based on the number of steps was moderate and averaged 7496.25 ± 7258.77. The following relationship was assumed for the number of steps calculated during activity performed each day at moderate intensity (average training heart rate): low physical activity < 5000–7499 steps, moderate physical activity 7500–9999 steps, and high physical activity > 10,000 steps (Table 3).

In the study group, 22.62% of the students did not do any supplementary physical activity, accounting for 69 respondents. Among the others, the amount of time spent on training averaged 2.24 h per week (134.4 min) and included such sports as running, swimming, fitness, martial arts, strength sports, and cycling.

To assess the normality of the distribution, the Kolomogorov–Smirnov test (a—Lilliefors significance correction) and the Shapiro–Wilk test were performed. The results of the tests indicated that the distribution of the variables is close to normal. The results of the variables were statistically significant (*p* < 0.05). Due to the results of the normality test of the distribution of variables, the rho–Spearman correlation coefficient test was used to tease out the correlation.

The Rosenberg scale measures body weight, BMI, and an average number of steps per week (*p* < 0.05). The result of the correlation of the Rosenberg scale with body weight and BMI showed a relationship. An increase in body weight and BMI will have a decreasing effect on the value obtained from the Rosenberg scale, and vice versa. In the case of the average number of steps per week, a higher average number of steps per week increases the Rosenberg scale score. No correlation was observed between the Rosenberg scale value and the number of workouts per week (Table 4.).

## 4. Discussion

Global self-assessment of students’ appearance and well-being is a widely researched topic. Research suggests that factors such as social pressures, appearance expectations, stress levels, and mental health affect students’ self-esteem. Overall, self-assessment of appearance and well-being varies among students around the world, depending on various cultural, social, and individual factors.

The main conclusion of the study is that an increase in BMI can impair global self-esteem. This is also significant in other scientific studies, which suggests that this conclusion can be taken as a certain regularity. Based on the available information, studies by teams such as Bogt et al. [21] and Ekeland et al. [22] suggest that people with higher body mass index (BMI) may tend to have lower self-esteem. A study by Mamazza et al. [23] underscores this, suggesting that black female students with higher BMIs may have particularly low self-esteem, especially about appearance. Remember, however, that the relationship between BMI and self-esteem is complex and can be determined by many other factors.

Other researchers have come to more specific conclusions. Analyzing the available research on students’ global self-esteem regarding external appearance, we note some significant differences. Tiggemann [24] conducted a study among Australian university students and found that many of them have low self-esteem related to appearance, which often leads to anxiety and stress. A study by Swami et al. [25] among students in the UK showed similar results, with additional gender differences. They found that women were more likely to have lower self-esteem about their appearance than men, which could affect their psychological well-being. In a study conducted by Chung and Choi [26] in Korea, they found that the influence of the media on students’ self-assessment of their appearance was significantly greater compared to previous studies. This indicates a global influence of culture and media on the self-assessment of appearance. A study by Bearman et al. [27] among American students found that negative attitudes toward one’s own body are strongly correlated with various health problems, such as eating disorders. Also, a study by Oishi and Kesebir [28] found that female students from countries with higher gender inequality indexes, such as India, Turkey, and Saudi Arabia, tended to have lower self-perceptions of their appearance than their counterparts in countries with lower inequality indexes, suggesting that gender inequality may affect self-perceptions of appearance. In addition, a study by Fardouly et al. [29] suggests that students’ exposure to idealized body images on social media may contribute to negative self-esteem regarding appearance.

It is worth noting that social media plays a key role in shaping responsibility and self-esteem. Through social media platforms, people learn to take responsibility for their statements, actions, and online interactions. Social media can also promote responsible behavior through social or educational campaigns. Social media has both positive and negative effects on users’ self-esteem. On the one hand, positive feedback and community support can boost self-confidence. On the other hand, comparison with others and negative comments can lower self-esteem. Therefore, it is important to use social media consciously and to be able to critically evaluate the content presented there [30].

Apart from the cultural and media influences mentioned earlier, socioeconomic factors can also play a role in students’ self-assessment of appearance and well-being. Research by Calmeiro and Gaspar de Matos [4] highlights that students from lower socioeconomic backgrounds may face additional challenges related to body image and self-esteem due to limited access to resources and opportunities. Addressing socioeconomic disparities and providing support to students from disadvantaged backgrounds could positively impact their self-esteem.

The school environment can significantly influence students’ self-esteem and body image. A study by Jones and Smolak [7] emphasizes the importance of promoting a positive and inclusive school culture that embraces diversity and body acceptance. Implementing school-based interventions and educational programs that foster body positivity and self-acceptance may contribute to improved self-esteem among students.

Family and peer relationships are crucial in shaping students’ self-perceptions. Research by Daniels and Gillen [8] suggests that parental support and communication about body image issues can have a protective effect on adolescents’ self-esteem. Similarly, peer support and positive social interactions can play a role in boosting self-confidence and well-being.

Given the complex relationship between mental health and self-esteem, providing mental health support and counseling services within educational settings is essential. A study by Mamazza et al. [23] highlights the significance of addressing mental health concerns, especially among students with higher BMI, to enhance their self-esteem and overall well-being.

The body positivity movement has gained traction globally, aiming to challenge beauty standards and promote body acceptance. Researchers such as Swami et al. [25] emphasize the potential of such movements in countering negative body image issues and improving self-esteem among students. Cooperation between educational institutions and body positivity advocates is crucial to creating an atmosphere of full acceptance and support among students. By promoting acceptance of one’s own body, we increase the self-confidence of young people, which has a direct impact on reducing the risk of mental problems such as depression and anxiety. It also acts as a prevention of bullying based on physical appearance. In addition, by accepting diversity, students learn healthy living habits and the ability to build strong interpersonal relationships. Ultimately, such cooperation prepares young people to live in a world full of diversity, teaching them to appreciate the value of each person [31].

In conclusion, the relationship between self-assessment of appearance and well-being among students is multi-faceted and influenced by various cultural, social, and individual factors. Encouraging physical activity, promoting body positivity, providing mental health support, and creating inclusive school environments are all important steps in fostering positive self-esteem and overall mental and emotional health among students. Continued research and collaboration among researchers, educators, and policymakers can lead to the development of effective interventions and strategies to support students’ well-being.

Comparing these results, students’ self-assessment of appearance is complicated and is influenced by many factors, such as culture, gender, and media influence. To improve students’ self-assessment of appearance, it is necessary to further study these factors and develop interventions that can help reduce negative influences. All these studies indicate a positive relationship between regular physical activity and levels of global self-esteem. Engaging in physical activity can contribute to increased self-esteem, self-confidence, and overall life satisfaction. It is worth encouraging and promoting physical activity across age groups to support an individual’s mental and emotional health.

Shaping self-esteem is important in the context of educating and raising young people, where values and ethics play a key role. Their study by Kobylarek et al. [32] points to the need to pay attention to the consequences that this decline in ethics can have on education and the formation of social attitudes. In light of the growing problem of sedentary lifestyles among young people, it is noted how important it is to promote physical activity among students [33].

### Strengths and Limitations

The study has several important strengths. The survey considers many different aspects—global self-esteem, body composition, and physical activity—which allows for a deeper understanding of these relationships. The study focuses on a specific population, which can provide valuable information for those working in the Polish education system, such as teachers, counselors, or psychologists. The study’s conclusions can help highlight the importance of physical activity for self-esteem and physical health, which can help promote healthier lifestyles. The results may provide valuable information for further research on the topic, as well as direct attention to potential areas that may require further exploration. The study may have implications for health policy, education, and intervention for students, emphasizing the importance of physical activity and healthy self-perception.

Our study has several potential limitations, based on the information that can be predicted from this type of study: its results may not be representative of other population groups, such as international students or non-students; students who chose to participate in the study may be more interested in the topic of physical activity and self-reporting, which may result in skewed results; physical activity is often difficult to measure accurately. If it is based on self-reporting, physical activity may be under- or overestimated; depending on how a study defines and measures “body composition”, different interpretive problems may arise. For example, some studies may focus on a body mass index (BMI) that does not distinguish between muscle and fat mass.

Limitations of testing with the Rosenberg Self-Assessment Scale include cultural differences, subjectivity, failure to distinguish between different aspects of self-assessment, state versus trait variability, and interpretation of results. The SES is developed based on Western norms and values and may not be as effective in different cultural contexts. The SES relies on subjective self-assessment, which may lead respondents to be incompletely honest due to a desire to present themselves in a positive light. In addition, it measures overall self-esteem but does not distinguish between different aspects of self-esteem. Self-esteem is also variable and depends on current mood and experiences, which the SES scale does not consider. Finally, there is no clear line between “low” and “high” self-esteem on the SES scale, interpreting the results as subjective and context-dependent. In addition, the stagnant state of affairs was helped by the COVID-19 pandemic [34], so this thread will be addressed in other studies by the authors.

## 5. Conclusions

Students who possess a more robust sense of global self-esteem often seem to be more inclined to participate in consistent physical activities. This inclination not only contributes to a healthier body composition but also reinforces their self-worth and confidence. On the flip side, those students who feel content with their body structure often exude a higher level of self-esteem. This satisfaction stems from a combination of factors, including societal perceptions, personal health choices, and the positive feedback loop created by a fit and active lifestyle.

Conversely, students who harbor dissatisfaction with their physical appearance or body composition might struggle with diminished self-esteem. This lack of confidence can deter them from participating in regular physical activities, further exacerbating the cycle of inactivity and negative self-perception. Such students might become trapped in this feedback loop, where their reluctance toward physical activity hinders any improvement in their body composition, leading to continued low self-esteem.

Regular engagement in physical activities has numerous benefits. Among these are the promotion of higher self-esteem and the development of a healthier body composition. When students are physically active, they are not only improving their physical health but also bolstering their mental and emotional well-being. The relationship between self-esteem, body satisfaction, and physical activity is multifaceted and cyclical, with each component influencing and being influenced by the others. Emphasizing the importance of this interconnectedness can guide educational and wellness programs aiming to enhance students’ overall well-being.

## Figures and Tables

**Table 1:** Body weight and BMI value in the group of students surveyed.

Variable	Numbers	Mean and SD	Median	Dominant	Min.	Max.
Body mass [kg].	n = 305	71.693 ± 13.729	68.90	62.0	48.2	115
BMI	n = 305	25.14 ± 4.277	24.24	20.96	17.63	42.07
BF [%].	n = 305	25.35 ± 4.27	18.4	32.6	13.5	45.0
LBM [%].	n = 305	74.64 ± 8.45	71.6	68	63	94.8

**Table 2:** Results of the Rosenberg scale in the group of students surveyed.

Variable	Numbers	Mean and SD	Median	Dominant	Min.	Max.
Rosenberg scale	n = 305	29.92 ± 5.182	32	33	20	39

**Table 3:** Evaluation of physical activity in the group of students surveyed.

Variable	Numbers	Mean and SD	Median	Dominant	Min.	Max.
Pedometric measurement	n = 305	7496.25 ± 7258.77	6270.0	5714	1456	14,018.6
Training [hour/week].	n = 305	2.24 ± 1.76	2.0	0	0	6

**Table 4:** Correlation values between Rosenberg scale and body weight, BMI, and physical activity in the group of students surveyed.

Spearman’s Rho		Body Weight	BMI	Average Number of Steps per Week	Number of Training Sessions per Week
Rosenberg scale	Correlation (*p*)	−0.309 **	−0.305 **	0.166	0.102
	Bilateral relevance	<0.001	<0.001	0.004	0.074
	n	305	305	305	305

**—score for statistical test.

## Data Availability

Not applicable.
